# Adrenocortical oncocytic neoplasms: a case report

**DOI:** 10.3389/fmed.2025.1709401

**Published:** 2025-11-03

**Authors:** Lintao Cao, Xiaoxiao Dong, Jiufeng Tan, Zhenpu Wang

**Affiliations:** ^1^The Fifth Clinical Medical College of Henan University of Chinese Medicine, Zhengzhou, Henan, China; ^2^Zhengzhou First People Hospital, Zhengzhou, Henan, China; ^3^Department of Urology, China-Japan Union Hospital of Jilin University, Changchun, Jilin, China; ^4^Provincial Key Laboratory of Molecular Diagnosis of Urological Tumors, Changchun, China; ^5^Jinlin Provincial Key Laboratory of Urological Tumors, Changchun, China

**Keywords:** adrenal cortical tumor, adrenocortical oncocytic neoplasms (ACONs), diagnosis, differential diagnosis, pathology

## Abstract

Adrenocortical oncocytic neoplasms (ACONs) are very rare in adrenal cortical tumors. One case was reported in this paper. During the physical examination, a soft tissue density shadow was about 6 cm × 5.7 cm in the right adrenal gland. A preoperative diagnosis was adrenal pheochromocytoma with concurrent laparoscopic resection of the right adrenal tumor. Postoperative pathological results rewarded the adrenal cortical oncocytic neoplasms ACONs. After 11-months of postoperative follow-up, the patient had no signs of tumor recurrence and related complications.

## 1 Introduction

Oncocytomas are epithelial tumors composed of cells with an eosinophilic, mitochondria-rich cytoplasm (1). Histologically, oncocytic tumors include large polygonal cells with both granular and eosinophilic cytoplasm. This characteristic cellular appearance can be attributed to the compensatory hyperplasia of mitochondria allegedly caused by chronic hypoxic injury (2). They can occur in various organs, such as the kidney, thyroid, pituitary, salivary gland, and parathyroid gland (3). However, adrenocortical oncocytic neoplasms (ACONs) are extremely rare. Since the first case was reported by Kakimoto et al. (4), approximately 200 cases have been reported in the literature. In 2004, Bisceglia et al. proposed diagnostic criteria for oncocytic neoplasms, classifying them into benign neoplasms, neoplasms with malignant potential, and malignant neoplasms (5). The most widely used Weiss scoring criteria are used to judge benign and malignant common adrenocortical tumors (6). Due to the scarcity of clinical cases, limited literature reports, and little information based on single experiences, this case report aims to contribute to the knowledge of adrenocortical oncocytoma.

## 2 Case presentation

### 2.1 Background of the case

The patient, a 42-year-old unmarried male, caught our attention due to paroxysmal hypertension. Two years ago, the patient developed intermittent elevated blood pressure without headache, sweating, and palpitations. The highest blood pressure was up to 170/100 mmHg. An enhanced abdominal computed tomography (CT) scan indicated a circular soft tissue density shadow in the right adrenal area, measuring approximately 6 cm × 5.7 cm, leading to a diagnosis of adrenal cortical adenoma. The local hospital recommended surgery, but the patient, who had a history of depression for 8 years, did not follow the advice during his treatment for depression. At the time of presentation, the patient was emotionally stable, but his blood pressure still fluctuated greatly. Moreover, he had no previous significant medical or family history.

### 2.2 Laboratory tests and imaging examination

**Figure 1:**
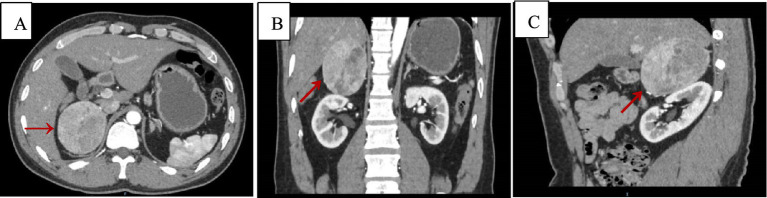
Adrenal CT + CTA scan, arterial phase images. Axial **(A)**, coronal **(B)**, and sagittal **(C)** views demonstrating a suprarenal mass measuring 6.6 cm × 5.2 cm × 7.0 cm, with a CT value of approximately 34 Hu (red arrows).

Upon admission, routine blood tests indicated a hemoglobin level of 93 g/L, and no abnormalities were found in adrenal-related hormones and residual tests. Adrenal CT + CT angiography showed a slightly lower density mass in the right adrenal gland, with a clear boundary, measuring approximately 6.6 cm × 5.2 cm × 7.0 cm, and a CT value of 34 HU (Figure 1).

### 2.3 Treatment

Based on the patient’s preoperative symptoms and imaging studies, the preoperative diagnosis was adrenal cortical adenoma. The patient underwent posterior laparoscopic resection of the right adrenal tumor through the retroperitoneal route. The intraoperative blood pressure was stable, and the surgical procedure was smooth. Hydro prednisone replacement therapy was given for three consecutive days post-surgery, and the postoperative blood pressure remained stable at 120–130/80–90 mmHg.

### 2.4 Outcome and follow-up

**Figure 2:**
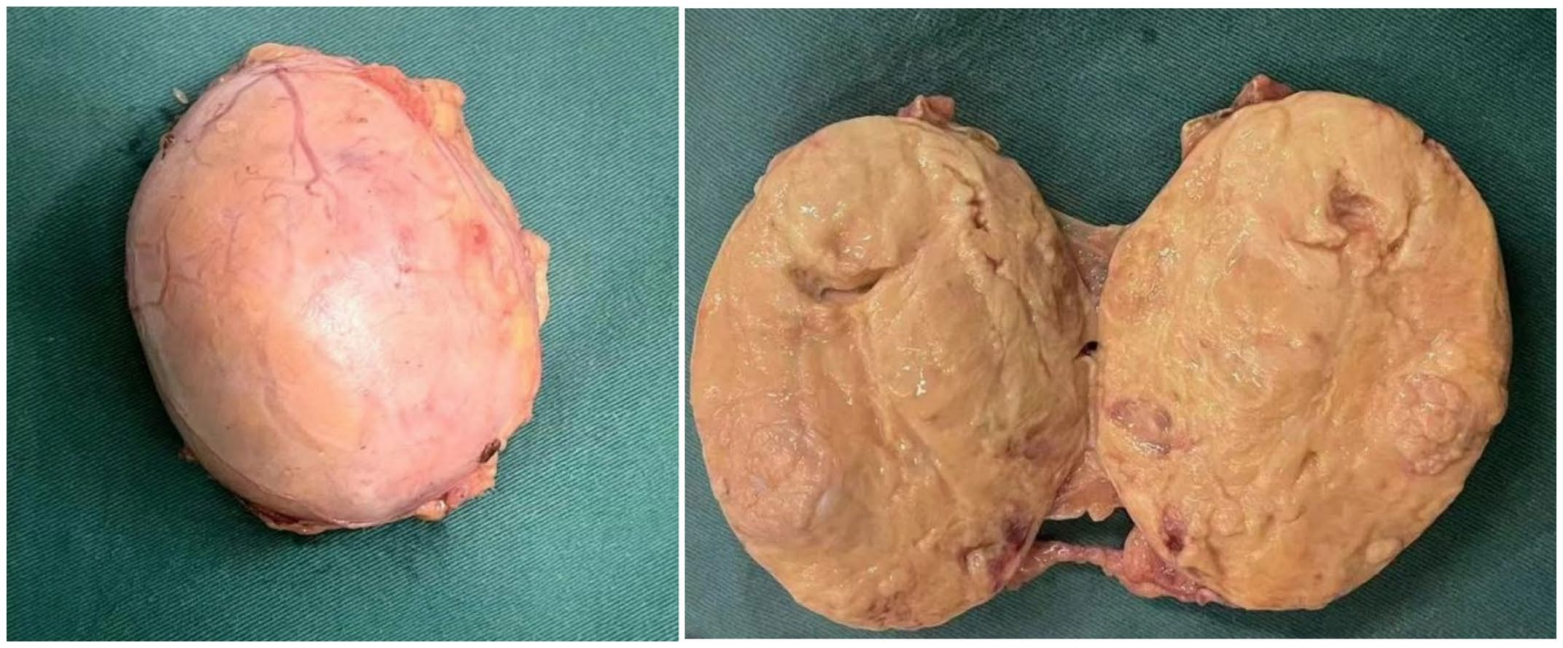
Postoperative specimen gross view: the surface capsule is smooth and intact, and the cut surface is grey-yellow solid.

**Figure 3:**
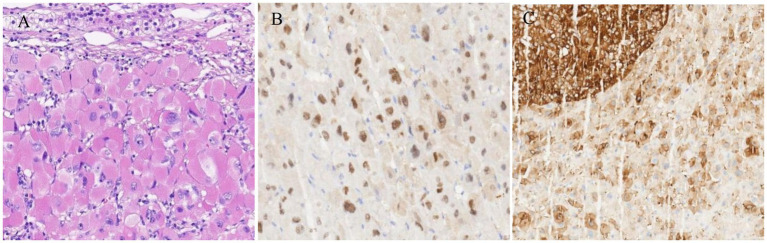
**(A)** Microscopic examination of the mass showed diffuse proliferation of polygonal cells with large irregular highly pleomorphic nuclei, prominent nucleoli, and abundant granular eosinophilic cytoplasm (HE, 400×). **(B,C)** Immunohistochemical staining of the resected adrenal tumor. **(B)** SF1 (+); **(C)** Syn (+).

The general view of postoperative specimens showed one nodule, measuring 7.5 cm × 6 cm × 6 cm, with a smooth and intact surface envelope (Figure 2). The cut surface was grey-yellow solid with a local capsule surface attached to a small amount of adrenal tissue. Postoperative pathology confirmed adrenal cortical eosinophilic tumors. Immunohistochemical staining revealed CK (pan) (−), Vimentin (−), GATA-3 (−), SF1 (+), Ki67 (1%+), Inhibinɑ (+), Syn (+), P53 (−), S-100 (−), CgA (−), CEA (−), EMA (−), *β*-Catenin (−) (Figure 3). After 11 months of postoperative follow-up, the patient had no associated complications and no signs of tumor recurrence.

## 3 Discussion

ACONs can occur at any age but are more frequent in women, with a male-to-female ratio of approximately 1:2 (7). Much like other adrenal masses, they are usually found incidentally, and are largely non-functional, lacking characteristic clinical manifestations. However, up to 20% of ACONs can secrete hormones such as cortisol, aldosterone, and androgens, occasionally leading to symptoms such as masculinization, precocious puberty, hypercortisolism, and primary hyperaldosteronism (5, 7, 8).

ACONs are typically large, averaging 8 cm in size, and most tumors have a complete capsule with non-invasive growth. The size and imaging findings (CT or MRI) of ACONs cannot reliably differentiate them from other adrenal tumors or distinguish them from malignant oncocytic tumors (1). Specific imaging features for oncotropic adrenocortical tumors have not been reported (9, 10). Therefore, they are often misdiagnosed as other adrenal tumors due to the lack of understanding. Thus, they need to be differentiated from the following diseases: (1) Adrenal pheochromocytoma; which has typical clinical manifestations and biochemical changes including significant blood pressure fluctuations, reaching over 200 mmHg, severe headaches, profuse sweating, palpitations, tachycardia, and other symptoms, as well as abnormal secretion of large amounts of catecholamines (norepinephrine, epinephrine, dopamine). CT imaging of adrenal pheochromocytomas reveals a large round or oval mass, with a diameter of ~3–5 cm. Large tumors often have an uneven density, and the solid part is obviously enhanced. The main pathological findings include a significant nest or trabecular structure. For slender blood vessels, large tumor cells, obvious nucleoli, cytoplasmic granulations, basophilia to dictrophilia, sometimes obvious tumor cells and nuclear pleomorphic can be seen. Immunohistochemistry for CgA (+), Melan-A (−), and supporting cells S-100 (+) may also be seen. (2) Adrenal metastases; in which case the patient may have a history of cancer which can involve both adrenal glands, rarely causing changes in adrenal function. The lesions had different morphology, no significant change in the MRI chemical shift reverse position check signal, and the enhancement scans showed uniform or heterogeneous enhancement. (3) Adrenocortical adenoma; in which case patients would present without special functional clinical manifestations. CT manifestations include a round or oval mass with smooth edges. Since 70% of adenomas are lipid-rich and have low density, often below 10 HU; dynamic contrast-enhanced examination shows that the mass is significantly enhanced and rapidly cleared, which is a characteristic feature. Specimen sections are typically yellow or brown. The main pathological features include cells arranged nests or bundles of bright and dark cells, with rich vascular and sinus-like structures. The cells are typically large and may have features of atypia but without capsule or vascular invasion. Notably, the approach to surgical approaches and postoperative monitoring is essentially the same as for other primary adrenal tumors.

The definitive diagnosis of ACONs relies on histopathological evaluation. Microscopically, these tumors consist of large polygonal cells with abundant eosinophilic and granular cytoplasm. Currently, the effect of immunohistochemical markers on the differentiation of good and malignant oncocytic tumors is still limited. Studies have reported that cases expressing Syn and cases not expressing vimentin are more likely to be benign (7). Most of the relevant studies and this study have shown that SF-1 is expressed, but its prognostic value in ACONs remains to be further analyzed. Other commonly used markers include Syn, Calretinin, CD56, CK (AE1/AE3), vimentin, etc. Adrenal cortical oncocytic tumors may express these markers to varying degrees, while CgA and S-100 are usually not expressed. At present, the role of immunohistochemical markers in distinguishing benign from malignant oncocytic tumors is still limited. Ki-67 proliferation index is an important indicator of cell growth activity and proliferation potential. Renaudin et al. (11) reported that Ki-67 cortical tumors ≤5% proliferation index had a good prognosis, and those with Ki-67 proliferation index >10% had a poor prognosis. In this study, the Ki-67 proliferation index was ≤5% in benign cases, and no recurrence tendency was observed during the follow-up period. The pathological diagnosis and postoperative behavioral characteristics of ACONs can be assessed using the Lin–Weiss–Bisceglia (LWB) system, the reticulin algorithm, or the Helsinki scoring system. However, the LWB criteria, proposed in 2004, are commonly used to differentiate between benign and malignant ACONs. The LWB criteria include three major criteria: (I) a mitotic rate >5 mitoses per 10 mm^2^ (50 high-power fields), (II) tumor necrosis, and (III) vascular invasion (angioinvasion). Additionally, there are four minor criteria: (I) large size (>10 cm and/or >200 g), (II) necrosis, (III) capsular invasion, and (IV) sinusoidal invasion. According to the LWB criteria, the pathological diagnosis of ACONs is as follows: Oncocytic adrenal cortical carcinoma: Presence of at least one major criterion. Oncocytic adrenal cortical neoplasm of uncertain malignant potential: Presence of at least one minor criterion. Oncocytic adrenal cortical adenoma: Absence of both major and minor criteria. In this case, neither primary nor minor criteria were observed, leading to the diagnosis of oncocytic adrenal cortical adenoma.

Comparison to adrenal cortical carcinoma shows that patients with benign or uncertain malignant potential ACONs have a favorable overall outcome. Furthermore, retrospective analysis indicates that adrenal cortical carcinoma is more significantly associated with a high Ki-67 index and a mitotic count greater than 5/50 high-power fields (7, 12). However, in the study by P. H. Graham, one patient with an oncocytic tumor with uncertain malignant potential developed distant metastasis at 53 months postoperatively, which may be associated with a high Ki-67 (13). This finding underscores the utility of Ki-67 and mitotic count in assessing malignancy grade and recurrence risk among various ACONs. Therefore, regular follow-up is required for patients with ACONs with uncertain malignant potential due to the risk of distant metastasis.

## 4 Conclusion

In conclusion, the diagnosis of ACONs mainly relies on histopathology and immunohistochemical detection, and the treatment is mainly surgical treatment. Moreover, given the borderline and malignant potential of some adrenal oncocytic tumors, close postoperative follow-up is necessary. Future studies should focus on accumulating more case reports and clinical data to better understand the behavior, prognosis, and optimal management strategies for ACONs.

## Data Availability

The original contributions presented in the study are included in the article/supplementary material, further inquiries can be directed to the corresponding authors.

## References

[1] Shah VN Premkumar A Walia R Kumar S Nahar U Bhansali A. Large but benign adrenal mass: adrenal oncocytoma. Indian J Endocrinol Metab. (2012) 16:469–71. doi: 10.4103/2230-8210.95717, PMID: 22629525 PMC3354866

[2] Canberk S Livolsi VA Baloch Z. Oncocytic lesions of the neuroendocrine system. Adv Anat Pathol. (2014) 21:69–82. doi: 10.1097/pap.0000000000000011, PMID: 24508690

[3] Smirnova EA Mikhaĭlov IG. Elektronno-mikroskopicheskaia kharakteristika onkotsitomy legkogo, tonkoi kishki, nadpochechnika. [Electron microscopic characteristics of oncocytoma of the lung, small intestine and adrenal gland]. Arkh Patol. (1986) 48:79–81. 3019283

[4] Kakimoto S Yushita Y Sanefuji T Kondo A Fujishima N Kishikawa M. Non-hormonal adrenocortical adenoma with oncocytoma-like appearances. Hinyokika kiyo. (1986) 32:757–63. 3751804

[5] Bisceglia M Ludovico O Mattia AD Ben-Dor D Sandbank J Pasquinelli G. Adrenocortical Oncocytic tumors: report of 10 cases and review of the literature. Int J Surg Pathol. (2004) 12:231–43. doi: 10.1177/106689690401200304, PMID: 15306935

[6] Weiss L. Comparative histologic study of 43 metastasizing and nonmetastasizing adrenocortical tumors. Am J Surg Pathol. (1984) 8:163–70. doi: 10.1097/00000478-198403000-00001 6703192

[7] Kanitra JJ Hardaway JC Soleimani T Koehler TJ MK ML Kavuturu S. Adrenocortical oncocytic neoplasm: a systematic review. Surgery. (2018) 164:1351–9. doi: 10.1016/j.surg.2018.04.044 30037428

[8] Zhouzhou Bao WH Di W Gao H. Hyperandrogenism caused by a rare adrenocortical oncocytic neoplasm with uncertain malignant potential: a case report and review of the literature. Endocr J. (2023) 70:275–80. doi: 10.1507/endocrj.Ej22-0277, PMID: 36384706

[9] Shah RK Oto A Ozkan OS Ernst RD Hernandez JA Chaudhary HB. Adrenal oncocytoma: us and Ct findings. JBR-BTR. (2004) 87:180–2. 15487257

[10] Park BK Kim B Ko K Jeong SY Kwon GY. Adrenal masses falsely diagnosed as adenomas on unenhanced and delayed contrast-enhanced computed tomography: pathological correlation. Eur Radiol. (2006) 16:642–7. doi: 10.1007/s00330-005-0065-5 16215735

[11] Renaudin K Smati S Wargny M Al Ghuzlan A Aubert S Leteurtre E. Clinicopathological description of 43 oncocytic adrenocortical tumors: importance of Ki-67 in histoprognostic evaluation. Mod Pathol. (2018) 31:1708–16. doi: 10.1038/s41379-018-0077-8, PMID: 29921900

[12] Shirali AS Zagzag J Chiang YJ Huang H Zhang M Habra MA. ASO visual abstract: differences in the Clinicopathologic behavior of Oncocytic adrenocortical neoplasms and conventional adrenocortical carcinomas. Ann Surg Oncol. (2022) 29:5555–63. doi: 10.1245/s10434-022-11626-w 35499784

[13] Kiernan CM. Commentary on “differences in the Clinicopathologic behavior of Oncocytic adrenocortical neoplasms and conventional adrenocortical carcinomas” by Shirali et al. Ann Surg Oncol. (2022) 29:5364–6. doi: 10.1245/s10434-022-11649-3, PMID: 35461425

